# A qualitative study of gestational weight gain goal setting

**DOI:** 10.1186/s12884-016-1118-2

**Published:** 2016-10-20

**Authors:** Shaniece Criss, Emily Oken, Lauren Guthrie, Marie-France Hivert

**Affiliations:** 1 Department of Health Sciences, Furman University, 3300 Poinsett Hwy, Greenville, SC 29613 USA; 2 Obesity Prevention Program, Department of Population Medicine, Harvard Medical School and Harvard Pilgrim Health Care Institute, 401 Park Drive Suite 401E, Boston, MA USA 02215

**Keywords:** Gestational weight gain, Goal-setting, Pregnancy, Body mass index

## Abstract

**Background:**

Gestational weight gain (GWG) is an important predictor of short and long-term pregnancy outcomes for both mother and child, and women who set a GWG goal are more likely to gain within recommended ranges. Little information is available regarding potentially modifiable factors that underlie a woman’s GWG goals. Our aims were to explore women’s perceptions regarding factors that affect GWG, their understanding of appropriate GWG, their goal-setting experiences including patient-health care provider (HCP) conversations, and supportive interventions they would most like to help them achieve the recommended GWG.

**Methods:**

We conducted nine in-depth interviews and seven focus groups with a total of 33 Boston, Massachusetts (MA) area women who were pregnant and had delivered within the prior 6 months. We recorded and transcribed all interviews. Two investigators independently coded resulting transcripts. We managed data using MAXQDA2 and conducted a content analysis.

**Results:**

Perceived factors that contributed to GWG goal-setting included the mother’s weight control behaviors concerning exercise and diet—including a “new way of eating for two” and “semblance of control”, experiences during prior pregnancies, conversations with HCPs, and influence from various information sources. Women focused on behaviors with consistent messaging across multiple sources of information, but mainly trusted their HCP, valued one-to-one conversations with them about GWG, preferred that the HCP initiate the conversation about GWG goals, and would be open to have the conversation started based on visual aid based on their own GWG progression.

**Conclusions:**

Pregnant women highly value discussions with their HCP to set GWG goals. Pregnant women view their clinicians as the most reliable source of information and believe that clinicians should open weight-related discussions throughout pregnancy.

## Background

Excessive gestational weight gain (GWG) is associated with adverse health outcomes in both mothers and children. In mothers, excessive GWG increases the risk of post-partum weight retention and thus accumulation of additional excess weight later in life [1–3]. Overweight women are at greater risk of excessive GWG, setting them doubly at risk of type 2 diabetes and other cardiovascular risk factors [4, 5]. Children born to mothers who experienced excessive GWG are more likely to have greater adiposity and adverse cardiometabolic profiles in childhood and later in life [6–12].

In 2009, the Institute of Medicine (IOM) published revised recommendations about the amount of GWG a woman should achieve according to her pre-pregnancy body mass index (BMI). GWG is an on-going challenging issue with 40 to 78 % of pregnant women gaining above the recommended range in various populations of pregnant women in the United States [13–15]. Determinants of GWG are multiple; one major factor is whether a woman has set a GWG goal for herself and whether that goal is within the recommended range [16]. Goal-setting is a key concept in behavioral approaches necessary in weight management. In a previous survey, we showed that only about 40 % of pregnant women had a goal consistent with IOM recommendations, another 20 % had discordant goals, and 40 % did not have a goal [17]. Those with no goal were more likely to gain outside the recommended amount of weight, either inadequate or excessive weight gain.

In this study, we focused on factors that contribute to GWG goal-setting of pregnant women. Our study is based on the Ecological Model for Health Promotion [18], which builds upon the social ecological model, to examine various levels of influence of GWG goal-setting: individual (e.g., personal attitudes), interpersonal (e.g., clinician interaction; family conversations), organizational (e.g., a GWG chart; availability of specialized health professionals for interventions), and community (e.g., media). The aims of this qualitative study using detailed interviews and focus groups in pregnant women receiving care at Atrius Harvard Vanguard Medical Associates, a large multi-center clinical practice in the greater Boston Area, USA were to:

1. Explore women’s perceptions of factors that affect GWG, including diet, exercise, and information sources and content;
2. Explore pregnant women’s understanding of appropriate GWG, GWG goal-setting, and weight control behavior;
3. Describe women’s experiences with health care providers (HCP) and advice they have received regarding GWG; and
4. Generate feedback about the development of tools or supportive interventions they would most like to help them achieve recommended GWG.

## Methods

### Recruitment

We recruited participants for this study from the greater Boston area in spring 2011 (Round 1) and fall 2014 (Round 2) for focus groups and interviews. We targeted women who were at least 16 weeks pregnant or who had delivered <6 months prior so that they recently had an opportunity to interact with their HCP around GWG goals and tracking.

In Round 1, we recruited participants by means of newspaper advertisement and posted flyers in obstetric waiting rooms. Interested women contacted the study research assistant by telephone or email. The research assistant confirmed eligibility, and scheduled the focus group or interview session. In Round 2, we included only pregnant women with pre-pregnancy BMI ≥25 kg/m^2^ to enrich our population with women with excess weight. Clinic staff informed study staff when an eligible woman had her appointment, and then recruitment would occur on-site in the obstetric waiting room after the appointment.

### Study design and sample

We conducted seven focus groups and nine individual interviews for a total of 33 participants. We conducted separate focus groups with participants who had normal pre-pregnancy BMI (two groups) and those who were overweight or obese (BMI ≥25 kg/m^2^,five groups). One study staff member conducted all of the individual interviews (Round 1, *n* = 9 participants) with eligible participants unable to attend a focus group, another conducted four focus groups (Round 1, *n* = 14 participants), and a third conducted three focus groups (Round 2, *n* = 10 participants). All interviewers were trained and experienced in qualitative interview techniques. The Harvard Pilgrim Health Care Human Studies Committee approved the study and all participants provided written informed consent.

### Interview guide

We developed the interview guide based on a review of the literature, Ecological Model for Health Promotion [18], and previous experience with this population. Table 1 shows the topic domains. We structured open-ended questions to determine: 1) insights about attitudes and behaviors about factors that affect GWG, 2) GWG goal-setting, 3) HCP interaction and advice about GWG, and 4) tools and resources for GWG management.

**Table 1:** Gestational weight gain goal setting interview guide domains and question examples

Domain	Question Examples
Diet related to pregnancy	How have you changed your diet since you found out that you are pregnant? Why have you made these changes?
	What makes it hard for you to eat the way you would like to be eating?
Physical activity related to pregnancy	What impact does your exercise have on your baby?
	What concerns do you have about risks related to exercising during pregnancy?
Gestational weight gain (GWG)	How important do you think the amount of weight you gain during pregnancy is for the health of your pregnancy?
	How much do you worry about your pregnancy weight gain?
Weight gain goal	What is your weight gain goal?
	How did you decide on this goal? Is this something you decided upon on your own or in consultation with your OB or someone else?
Patient-Clinician Conversations	Has your OB discussed weight gain during pregnancy with you? How and when did s/he raise the topic?
	During the course of this pregnancy, have any of your OB providers told you whether you are gaining about the right amount, too much, or too little?
Information sources	Where have you heard information about weight gain during pregnancy? How much do you trust this source?
Weight control behavior	How much control do you feel that have over how much weight you gain during pregnancy? What factors make you feel that you are not in control?
	Are you actively trying to influence the amount of weight you gain during your pregnancy? What are you doing to influence your weight gain?
Tools and resources for GWG management	Imagine that you were gaining too much weight during your pregnancy. What do you think would be most helpful for you to help you get back on track?
	How would you feel about seeing a graph of your weight gain during pregnancy? Who would you like to get this graph from, your doctor, nurse, midwife, or someone else?
	Have you used pregnancy-related applications on your smart phone? Which ones? What do you like about them? What do you use the apps for?

### Data collection and analysis

All interviews and focus groups were conducted at a central Boston clinic location, and lasted 60 to 90 min. We conducted focus groups in the evening to maximize participation, and provided dinner. Participants received a $25 gift card and transportation reimbursement.

To protect the privacy of participants, we made every effort to capture audio recordings with no identifying information. All audio recordings were digitally recorded, transferred to a secure computer, transcribed, and de-identified. Members of the research team immersed themselves in the data, coded, identified themes, reviewed the results, and continued discussion until a consensus was met [19]. Specifically, a study team member read the transcripts to get an overall impression of potential themes, and developed the codebook based on the interview guide and the major themes identified in each of the transcripts. Three reviewers evaluated the codebook to ensure accuracy and clarity of code definitions. Round 1 transcripts were then independently coded by two reviewers using MAXQDA2, a qualitative software management program. In addition, a third reviewer coded and completed a content analysis on Round 1 and Round 2 transcripts.

## Results

The participants (*n* = 33) were on average 34 years old and at a mean of 23 weeks of gestation. Two-thirds of participants were of European descent, 15 % were African-American, and the remainder from other ethnic backgrounds. More than 80 % (27 out of 33) had completed a college degree or higher level of education. About half of the participants (17 out of 33) reported having pre-pregnancy BMI ≥ 25 kg/m^2^. The following themes were consistent among all groups, unless otherwise indicated.

### Perceived factors that affect GWG

#### Diet

##### A new way of eating for two

Many women shared stories of friends and family urging them to “eat for two,” yet they did not feel that they needed to overindulge in eating. Here is how one participant revised the concept:

“Because now I’m not only eating for myself, I’m eating for two, only not quantity-wise. Every time I say that I always think of people saying, ‘Oh, you’re eating for two. You can indulge.’ And I say, ‘Yes, technically I am.’ But it doesn’t mean that I’m going to go overboard. Or go and buy snacks that I wish I could have, but I know I can’t.”

Several women indicated that they felt that what they ate affected the baby: “I mean, you have to feed yourself and remember that also goes to the baby. If you’re eating crap now, you’re giving that crap to the baby.” Some women with previous pregnancies indicated that their child likes to eat what they ate in pregnancy. In contrast, some women did not think their current eating would cause any issues for the baby, and nutrition was important once s/he is born. Participants indicated barriers that prevented them from having a nutritious diet, including cost of healthy food, lack of time for food preparation (e.g., taking care of another child), not feeling like cooking, and the continuous availability of unhealthy food.

#### Physical activity

Several women reported that physical activity was important, but it was more challenging during pregnancy. One participant said, “The second I got pregnant, my first trimester, I went to the gym, and my inner body alarm clock was just screaming at me- don’t do it.” Most women reported that they were not currently exercising during pregnancy, although those who were participated in a variety of activities ranging from walking to belly dancing.

##### Exercise for mom, not baby

Many women did not see a clear link between physical activity and the health of the baby, but many reported that exercise could ease labor and enhance their own mental/emotional health. One participant said, “I pay more importance to diet, and I think exercise is just more important for myself, like for my feeling good and like, but I don’t think it affects baby much.” A few women said that modeling exercise to their born child would be more important.

The women listed numerous barriers to physical activity in pregnancy, including: reduced energy levels/being tired, trouble breathing, pain, lack of time due to professional and/or personal life (e.g., other children), inclement weather being a safety concern (e.g., falling on ice), and the expense of exercise classes.

### Understanding appropriate GWG, GWG goal-setting, and weight control behavior

#### Appropriate GWG

Many women shared the belief that excessive GWG can increase risks of gestational diabetes and preeclampsia, and that women need to closely monitor their weight if they have gestational diabetes. A few participants indicated that if the mother gained too much weight, then the child could be overweight or obese. Several participants were unable to identify specific health-related issues for the baby resulting from excessive GWG. In contrast, many participants reported that gaining too little weight is an issue because underweight babies can have health problems.

#### Weight gain goals

Several participants set GWG goals and reported ranges between 15 to 49 lb. Several women, including participants with BMI ≥25 kg/m^2^, stated that they did not have a GWG goal and they assumed that their HCP would discuss GWG with them if it was an issue: “I don’t have a goal. I just, I’ve gained already 35 lb, and I still have 2 more months left.” Many participants reported that they felt “conscious” of their weight. One woman said, “oh my gosh- my body’s changing so much. So for me, it’s already a little bit weird to be gaining weight like this. I’m always thinking, OK, you don’t wanna be gaining too much.” Other participants were not as concerned about weight gain because they felt that they would be able to “shed the pounds” post-partum based on losing weight with previous pregnancies or motivation to be active for their child.

#### Weight control behavior

##### Semblance of control

Most women reported that they felt as though they had “some” control over their weight gain, but the control varied based on needing more calories for pregnancy, mother and baby’s cravings, stress, trimester, and the way the body processes the nutrients. One participant said:

“I feel like I have some semblance of control…I feel like I could be more controlling of how much weight I was gaining if I weren’t pregnant. So I could be like, ‘Well, you know, I feel like I gorged the last two days so I’ll kind of chill out and just eat fruits and salads for the next couple of days.’ But I feel like I’d be cheating my baby if I did that right now.”

Concerning cravings, a participant said, “I have some control, but not much. Because sometimes I feel hungry and I’ve wanted some specific food that it’s hard to resist…you should eat it, because it means you lack something in the food.” Similarly, a woman stated, “If I feel the urge to eat… I kind of feel like that’s the baby that wants more. And I feel like I don’t want to deprive the baby of eating more.” One participant indicated that she had control and said, “I know ultimately it’s my fault. I cannot blame anyone else for my weight gain.” In contrast, another participant expressed she lacked control because heartburn and indigestion limited her options.

### Patient-HCP conversations and advice regarding GWG

#### Provider conversations

Most participants reported that they highly ranked their HCP’s advice concerning GWG. Specifically, the participants stated that they “trusted” their clinician. One participant said, “I trust my doctor more because she works with patients every day.” Another participant said, “If my doctor said, ‘This is what you have to do,’ then I would do it.” Participants reported receiving information about GWG in different ways, which is highlighted in the next sections.

##### Patient-initiated

Many participants reported that they specifically had to ask their HCP about appropriate weight gain based on curiosity or a prior health issue (e.g., diabetes), but that their HCP did not often offer this information spontaneously. Women also reported that they assumed that “no news was good news” if the doctor or midwife did not discuss weight. One participant reported, “So I sort of think, in general, unless you have a specific question, it doesn’t tend to come up in conversation.” Some participants did not ask their clinicians about weight gain because they thought their obstetrician or midwife would discuss weight gain with them if it was an issue. Several participants reported that discussing weight gain guidelines in the beginning of the first trimester would have been helpful.

##### HCP-initiated

Participants reported that HCP-initiated conversations typically stemmed from the patient being overweight or having health issues. One woman said, “Yeah, it [goal weight] came up pretty immediately. Because I was overweight when I got pregnant…other than that, it hasn’t been a drastic weight gain, so no one’s been talking about it.” Some participants shared that their HCP gave the weight recommendation during an early appointment. Throughout their appointments, several participants would say that the HCP would say that they’re “on track.” Several participants commented that if they heard their HCP advise them about their weight gain, then it would be an “impetus for actually changing something.”

##### HCP advice

Several women expressed that their provider has given recommendations about nutrition, physical activity, and/or weight gain, but the HCP did not explain why it was an issue or how to make the change. One participant stated, “[the doctor] never told me what I should be eating-- just to make sure that I was eating enough. But I don’t know what ‘enough’ is.” Another woman stated, “too much weight gain doesn’t mean anything to me. If there were vital signs…that were showing that my body was having a hard time [then my provider should explain].”

Participants gave examples of HCP advice they liked: “We want to see the baby grow, we don’t want to see you grow” and “feed yourself--think about it as if the baby’s sitting in front of you…it’s sort of stuck in my head (laughs). So now when I eat a 4^th^ Girl Scout cookie, I think, would I really shove that in a kid’s face? Probably not.” Some women reported that they did not like the way the provider gave weight-related advice: “the WIC lady that I see has said I’m like above the norm…who says that to a woman? It’s not helpful, especially because I’m pregnant.”

##### Referral to nutritionists

Most women reported that they would be open to meet with a nutritionist, especially if excessive GWG was an issue. Some participants stated that would prefer a nutritionist to talk about diet because they “studied” this area. Some women who had visited a nutritionist said that the information, ideas, and recipes were helpful. A few participants did not think the nutritionist’s visit was helpful because the person just handed out pamphlets or had an unpleasant demeanor. One person mentioned that her WIC nutritionist was “just not therapeutic…she just says her opinion and then moves on [and] doesn’t really have a discussion or anything.”

### Tools and resources for GWG management

#### Information sources

The main information sources for participants about GWG were HCP, family, friends, pregnancy websites, books, and magazines. Most participants reported that the HCP’s recommendations were the most trusted source. Participants reported that family would either say to “eat what they want” or “not to gain too much weight.” For instance, one woman stated, “my mom also told me [when I first got pregnant], ‘don’t get fat. There’s no need to get fat’” and another participant shared that her mother had said, “So-and-so gained hardly any weight. She’s really skinny. And all her kids are fine. I’m like, ‘Mom. Not helpful’.” Participants reported that their friends would typically be encouraging about their appearance and would give them helpful advice about their pregnancy, like “try to lose a little weight before pregnancy” or “I had gestational diabetes, so be careful.” Several participants reported reading pregnancy websites, apps, and weekly emails. Women trusted pregnancy-related websites from national medical organizations, but most did not trust comment sections on such websites or Facebook.

#### Potential interventions

Most participants said that they would be willing to attend information sessions to learn more about GWG if accessible and available at different times of the day. Some participants reported preferring group nutrition/cooking and exercise classes (e.g., walking in the park; yoga). Other participants indicated they would prefer more personalized attention. Several participants reported that they would be amendable to a health coach emailing and texting them, but felt that women with high-risk pregnancies would be better candidates for real-time conversations. Most participants reported receiving packets and would prefer to have a discussion with their HCP about the information because “I don’t think handing people packets [handouts] is educating them at all,” and reported that discussing weight gain guidelines in the beginning of the first trimester would be helpful, including that pregnant women only need an additional 300 calories a day. Some participants with BMI >25 kg/m^2^ stated that a handout with specific daily meal plans for pregnant women for breakfast, lunch, and dinner “would be perfect.”

#### GWG Chart

During the interviews and focus groups, we shared with the participants a chart that gives a visual display of GWG progression aligned with the IOM recommendation. Nearly all participants said that it was “useful” and “helpful.” Some people said the graph would be a good discussion starter and motivate them to modify their food intake and exercise; whereas, a few people said that they would not “pay too much attention to it,” they would not “want to know the number,” and they would “not want another piece of paper.” A few women requested that the GWG chart be available on the patient online system.

## Discussion

Using the McLeroy et al.’s ecological model [18], we elucidated the nuances associated with GWG and goal-setting for weight gain. Our qualitative analyses of semi-structured interviews and focus groups in pregnant women showed that factors that might influence GWG include mother’s beliefs concerning diet and exercise and experience during prior pregnancies (individual), conversations with HCPs (interpersonal), interest in clinics utilizing GWG charts (organizational), and influence of information sources (community). Women focused on behaviors that messages were consistent across multiple sources of information, but mainly trusted their HCP. Women highly valued one-to-one conversations with their HCP about GWG, especially if they have weight issues prior or during pregnancy.

### Implications for Practice and/or Policy

Indeed, one of the major points that we highlighted in our observations is that pregnant women rank highly clinician advice concerning GWG. Many points throughout the interviews and focus groups supported this conclusion. First, women considered clinicians the most reliable source of information and verified with their provider information found from other sources. This is in line with qualitative studies where women also reported that clinicians were the most credible source of information [20, 21], and a quantitative study that found that discordant clinician advice was directly associated with excessive GWG [22]. In addition, clinician input seems to also be an important adjunct to the use of educational tools about GWG. Women in our study reported having received many handouts, but felt that was not sufficient per se and that their providers need to discuss the content with them if the specific matter is an important health issue for the pregnancy. Similarly, pregnant women from a large academic medical center in Wisconsin also reported receiving handouts for nutritional education during pregnancy but underlined the fact that the material was rarely reviewed during the medical encounter [23]. In our study, when we presented a graphic tool to chart GWG during pregnancy, women stated that the tool would be useful if accompanied by a discussion with their provider, highlighting once again the importance of clinician’s input about GWG in women’s opinion.

We and others have shown that women whose provider recommended weight gains consistent with IOM recommendations were more likely to have a concordant GWG goal compared to women whose providers recommended gains inconsistent with recommendations or no clear GWG recommendations [17, 24]. Previous interviews found that most clinicians reported discussing appropriate GWG with their patients at the beginning of pregnancy [25]. Many women reported receiving a GWG goal, but this was not consistent across all participants; unfortunately, many overweight women reported not receiving a specific GWG goal. Similarly, Stengel et al. reported that nine out the 24 overweight/obese women they interviewed were not given a goal, and many were given vague goals or goals outside the IOM recommendations [21].

In the current analysis, women stated that they would like their providers to open the conversation and give them a goal for GWG early in pregnancy. In our previous qualitative study among obstetric providers, clinicians reported that they felt that weight is a sensitive issue for women and often wait for women to ask questions – or only bring up weight gain as a topic if GWG reaches out of the expected range [25]. This HCP behavior seems to be fairly common in obstetric providers since the theme also emerged from a systematic review in the UK [26] and in other US practitioners’ qualitative studies on GWG [27, 28]. Women in our study and in other reports [23, 29] felt that GWG should be addressed up front by their provider if this is important and not wait for women to bring up the topic of weight gain to the providers, an approach sometimes called ‘reactive’ counseling [23, 28]. It is striking that both parties feel like the other one should open the conversation: clinicians believe that weight is sensitive and usually wait until women raised the issue or when GWG was concerning, and women felt as though their provider should and would raise that discussion if that is something that is important for their health.

Providers’ confidence level in their own counseling skills is associated with higher adherence to guidelines for prenatal clinical care of obese pregnant women [30]. Consequently, the clinicians’ difficulty in opening the discussion might also derive from the fact that providers do not feel adequately prepared and feel uncertain about the impact of their counseling effort, as we and others reported based on interviews with obstetric providers [25, 27, 28, 31]. This situation is not unique to clinicians in obstetric care, since a few studies have reported that physicians often feel ill-prepared and lack confidence to provide adequate lifestyle counseling, particularly in the fields of physical activity, nutrition, and weight management [32, 33]. Multidisciplinary approaches and referral to appropriate health professionals are likely part of adequate weight management in obese women during pregnancy, but providers need to have confidence in their knowledge and skills about lifestyle counseling to open the discussions.

### Strengths and limitations

Strengths of our study include the use of standardized moderator guides, recording of the group discussions and interviews, and analyzing the transcripts using established qualitative methods. Our study also has limitations. We interviewed women of diverse race/ethnicity in the greater Boston, MA area, and most had college or graduate degrees, so results may not be generalizable to pregnant women living elsewhere or with lower education. In order to build upon this study, future studies will include a quantitative survey on a larger population. GWG goals are based on self-report and may have been over or underreported. Women who participated in the study are likely interested in weight related issues and so our results might overestimate the frequency of GWG goal-setting.

## Conclusion

Pregnant women highly value advice and the information about GWG provided by their HCP. Women hope to receive goals for appropriate GWG early in pregnancy from their primary HCP. To achieve the set GWG goals, women focused on behaviors that were consistently promoted from different sources of information, but mainly if the information was provided or confirmed by their HCP. Women believed that HCPs should proactively address GWG and related behaviors along the course of pregnancy – not just in a ‘reactive’ fashion. Based on our study and other studies [20–22, 29], it seems important to find ways to facilitate early conversations about GWG between pregnant women and HCP in clinical settings.

## Abbreviations

BMI: Body mass index
GWG: Gestational weight gain
HCP: Health care provider
IOM: Institute of Medicine.
